# Evaluation of the Lung Cancer Risks at Which to Screen Ever- and Never-Smokers: Screening Rules Applied to the PLCO and NLST Cohorts

**DOI:** 10.1371/journal.pmed.1001764

**Published:** 2014-12-02

**Authors:** Martin C. Tammemägi, Timothy R. Church, William G. Hocking, Gerard A. Silvestri, Paul A. Kvale, Thomas L. Riley, John Commins, Christine D. Berg

**Affiliations:** 1 Department of Health Sciences, Brock University, St. Catharines, Ontario, Canada; 2 School of Public Health, University of Minnesota, Minneapolis, Minnesota, United States of America; 3 Marshfield Clinic, Marshfield, Wisconsin, United States of America; 4 Pulmonary and Critical Care Medicine, Medical University of South Carolina, Charleston, South Carolina, United States of America; 5 Pulmonary and Critical Care Medicine, Henry Ford Health System, Detroit, Michigan, United States of America; 6 Information Management Systems, Rockville, Maryland, United States of America; 7 Department of Radiation Oncology and Molecular Radiation Sciences, Johns Hopkins Medicine, Baltimore, Maryland, United States of America; University of Illinois, United States of America

## Abstract

**Background:**

Lung cancer risks at which individuals should be screened with computed tomography (CT) for lung cancer are undecided. This study's objectives are to identify a risk threshold for selecting individuals for screening, to compare its efficiency with the U.S. Preventive Services Task Force (USPSTF) criteria for identifying screenees, and to determine whether never-smokers should be screened. Lung cancer risks are compared between smokers aged 55–64 and ≥65–80 y.

**Methods and Findings:**

Applying the PLCO_m2012_ model, a model based on 6-y lung cancer incidence, we identified the risk threshold above which National Lung Screening Trial (NLST, *n = *53,452) CT arm lung cancer mortality rates were consistently lower than rates in the chest X-ray (CXR) arm. We evaluated the USPSTF and PLCO_m2012_ risk criteria in intervention arm (CXR) smokers (*n = *37,327) of the Prostate, Lung, Colorectal and Ovarian Cancer Screening Trial (PLCO). The numbers of smokers selected for screening, and the sensitivities, specificities, and positive predictive values (PPVs) for identifying lung cancers were assessed. A modified model (PLCO_all2014_) evaluated risks in never-smokers. At PLCO_m2012_ risk ≥0.0151, the 65th percentile of risk, the NLST CT arm mortality rates are consistently below the CXR arm's rates. The number needed to screen to prevent one lung cancer death in the 65th to 100th percentile risk group is 255 (95% CI 143 to 1,184), and in the 30th to <65th percentile risk group is 963 (95% CI 291 to −754); the number needed to screen could not be estimated in the <30th percentile risk group because of absence of lung cancer deaths. When applied to PLCO intervention arm smokers, compared to the USPSTF criteria, the PLCO_m2012_ risk ≥0.0151 threshold selected 8.8% fewer individuals for screening (*p<*0.001) but identified 12.4% more lung cancers (sensitivity 80.1% [95% CI 76.8%–83.0%] versus 71.2% [95% CI 67.6%–74.6%], *p<*0.001), had fewer false-positives (specificity 66.2% [95% CI 65.7%–66.7%] versus 62.7% [95% CI 62.2%–63.1%], *p<*0.001), and had higher PPV (4.2% [95% CI 3.9%–4.6%] versus 3.4% [95% CI 3.1%–3.7%], *p<*0.001). In total, 26% of individuals selected for screening based on USPSTF criteria had risks below the threshold PLCO_m2012_ risk ≥0.0151. Of PLCO former smokers with quit time >15 y, 8.5% had PLCO_m2012_ risk ≥0.0151. None of 65,711 PLCO never-smokers had PLCO_m2012_ risk ≥0.0151. Risks and lung cancers were significantly greater in PLCO smokers aged ≥65–80 y than in those aged 55–64 y. This study omitted cost-effectiveness analysis.

**Conclusions:**

The USPSTF criteria for CT screening include some low-risk individuals and exclude some high-risk individuals. Use of the PLCO_m2012_ risk ≥0.0151 criterion can improve screening efficiency. Currently, never-smokers should not be screened. Smokers aged ≥65–80 y are a high-risk group who may benefit from screening.

*Please see later in the article for the Editors' Summary*

## Abstract 2

Martin Tammemägi and colleagues evaluate which risk groups of individuals, including nonsmokers and high-risk individuals from 65 to 80 years of age, should be screened for lung cancer using computed tomography.

*Please see later in the article for the Editors' Summary*

## Editors' Summary

**Background:**

Lung cancer is the most commonly occurring cancer in the world and the most common cause of cancer-related deaths. Like all cancers, lung cancer occurs when cells acquire genetic changes that allow them to grow uncontrollably and to move around the body (metastasize). The most common trigger for these genetic changes in lung cancer is exposure to cigarette smoke. Symptoms of lung cancer include a persistent cough and breathlessness. If lung cancer is diagnosed when it is confined to the lung (stage I), the tumor can often be removed surgically. Stage II tumors, which have spread into nearby lymph nodes, are usually treated with surgery plus chemotherapy or radiotherapy. For more advanced lung cancers that have spread throughout the chest (stage III) or the body (stage IV), surgery is rarely helpful and these tumors are treated with chemotherapy and radiotherapy alone. Overall, because most lung cancers are not detected until they are advanced, less than 17% of people diagnosed with lung cancer survive for five years.

**Why Was This Study Done?:**

Screening for lung cancer—looking for early disease in healthy people—could save lives. In the US National Lung Screening Trial (NLST), annual screening with computed tomography (CT) reduced lung cancer mortality by 20% among smokers at high risk of developing cancer compared with screening with a chest X-ray. But what criteria should be used to decide who is screened for lung cancer? The US Preventive Services Task Force (USPSTF), for example, recommends annual CT screening of people who are 55–80 years old, have smoked 30 or more pack-years (one pack-year is defined as a pack of cigarettes per day for one year), and—if they are former smokers—quit smoking less than 15 years ago. However, some experts think lung cancer risk prediction models—statistical models that estimate risk based on numerous personal characteristics—should be used to select people for screening. Here, the researchers evaluate PLCO_m2012_, a lung cancer risk prediction model based on the incidence of lung cancer among smokers enrolled in the US Prostate, Lung, Colorectal and Ovarian Cancer Screening Trial (PLCO). Specifically, the researchers use NLST and PLCO screening trial data to identify a PLCO_m2012_ risk threshold for selecting people for screening and to compare the efficiency of the PLCO_m2012_ model and the USPSTF criteria for identifying “screenees.”

**What Did the Researchers Do and Find?:**

By analyzing NLST data, the researchers calculated that at PLCO_m2012_ risk ≥0.0151, mortality (death) rates among NLST participants screened with CT were consistently below mortality rates among NLST participants screened with chest X-ray and that 255 people with a PLCO_m2012_ risk ≥0.0151 would need to be screened to prevent one lung cancer death. Next, they used data collected from smokers in the screened arm of the PLCO trial to compare the efficiency of the PLCO_m2012_ and USPSTF criteria for identifying screenees. They found that 8.8% fewer people had a PLCO_m2012_ risk ≥0.0151 than met USPSTF criteria for screening, but 12.4% more lung cancers were identified. Thus, using PLCO_m2012_ improved the sensitivity and specificity of the selection of individuals for lung cancer screening over using UPSTF criteria. Notably, 8.5% of PLCO former smokers with quit times of more than 15 years had PLCO_m2012_ risk ≥0.0151, none of the PLCO never-smokers had PLCO_m2012_ risk ≥0.0151, and the calculated risks and incidence of lung cancer were greater among PLCO smokers aged ≥65–80 years than among those aged 55–64 years.

**What Do These Findings Mean?:**

Despite the absence of a cost-effectiveness analysis in this study, these findings suggest that the use of the PLCO_m2012_ risk ≥0.0151 threshold rather than USPSTF criteria for selecting individuals for lung cancer screening could improve screening efficiency. The findings have several other important implications. First, these findings suggest that screening may be justified in people who stopped smoking more than 15 years ago; USPSTF currently recommends that screening stop once an individual's quit time exceeds 15 years. Second, these findings do not support lung cancer screening among never-smokers. Finally, these findings suggest that smokers aged ≥65–80 years might benefit from screening, although the presence of additional illnesses and reduced life expectancy need to be considered before recommending the provision of routine lung cancer screening to this section of the population.

**Additional Information:**

Please access these websites via the online version of this summary at http://dx.doi.org/10.1371/journal.pmed.1001764.

- The US National Cancer Institute provides information about all aspects of lung cancer for patients and health-care professionals, including information on lung cancer screening (in English and Spanish)
- Cancer Research UK also provides detailed information about lung cancer and about lung cancer screening
- The UK National Health Service Choices website has a page on lung cancer that includes personal stories
- MedlinePlus provides links to other sources of information about lung cancer (in English and Spanish)
- Information about the USPSTF recommendations for lung cancer screening is available

## Introduction

The National Lung Screening Trial (NLST) demonstrated that annual low-dose computed tomography (LDCT) screening reduces lung cancer mortality by 20% when applied to high-risk smokers (age 55–74 y, ≥30 pack-years, and <15 y of quit time [for former smokers, time since ceasing smoking]) [1]. Consequently, several institutions have recommended LDCT lung cancer screening of high-risk populations [2]–[7], and many health-care institutions have started or are planning LDCT screening programs. Most recommendations and programs rely on NLST risk criteria or variants of these criteria for selecting individuals for screening [8]. The U.S. Preventive Services Task Force (USPSTF) recommends annual screening of high-risk individuals, i.e., those who are 55–80 y, have smoked ≥30 pack-years, and have <15 y of smoking quit time [9]. Some of these criteria, which are similar to the NLST criteria, were based on microsimulation models developed by the Cancer Intervention and Surveillance Modeling Network (CISNET) lung group [10]. However, it has been shown that selecting individuals for screening based on accurate lung cancer risk prediction models is significantly more sensitive in detecting individuals who will be diagnosed with lung cancer and would save more lives than using the NLST criteria [11],[12]. Important issues regarding selection of individuals for lung cancer screening remain. It is unclear at what risk individuals should be screened, how efficient the USPSTF criteria are compared to model-based risk criteria, and into what risk threshold USPSTF recommendations translate.

Never-smokers have been excluded from lung cancer screening trials and programs, but this has not been based on quantitative evidence. Lung cancer in never-smokers is a major public health problem, accounting for approximately 10%–15% of lung cancers, and if considered separately, would rank seventh as a cause of cancer death [13],[14]. Although a survey found that a sizeable proportion of never-smokers would consider computed tomography (CT) screening [15], it has not been demonstrated that never-smokers can be at high enough risk to warrant screening.

In the United States on April 30, 2014, the Centers for Medicare & Medicaid Services convened the Medicare Evidence Development & Coverage Advisory Committee (MEDCAC) to evaluate the use of LDCT lung cancer screening in the Medicare population, primarily aged 65 y and older [16]. The MEDCAC panel gave low confidence scores for screening, and if its recommendation is followed, Medicare will not reimburse the cost of lung cancer screening in those 65 y and older. In contrast, because the USPSTF gave LDCT lung cancer screening a “B recommendation” in favor of screening high-risk individuals, the Patient Protection and Affordable Care Act will lead to reimbursement for screening of high-risk individuals aged 55–64 y. The impact of these discordant strategies is unclear.

In the current study, we extend evaluation and application of our PLCO_m2012_ model [11]. It is a logistic regression lung cancer risk prediction model based on 6-y incidence of lung cancer occurring in smokers in the control arm of the Prostate, Lung, Colorectal and Ovarian Cancer Screening Trial (PLCO). The model consists of four smoking variables (smoking intensity, smoking duration, quit time in former smokers, and current smoking status [current versus former]) and seven non-smoking variables (age, race/ethnicity, socioeconomic circumstance estimated by education level, body mass index, personal history of cancer, chronic obstructive pulmonary disease, family history of lung cancer). The PLCO_m2012_ model demonstrated high predictive performance, both discrimination and calibration, in external validation in PLCO intervention arm smokers.

This study further analyzes data from two major screening trials, the NLST and PLCO. Our study aims are as follows: (1) identify a risk threshold for selecting lung cancer screenees based on the PLCO_m2012_ [11] risk at which mortality rates in the NLST CT screening arm are consistently lower than those in the chest X-ray (CXR) screening arm; (2) compare performance of USPSTF versus PLCO_m2012_ risk criteria for selecting screenees, based on lung cancer incidence and mortality; (3) as an alternate PLCO_m2012_ risk threshold, estimate the PLCO_m2012_ risk that selects a proportion of smokers equal to that selected by USPSTF criteria; (4) determine whether high-risk never-smokers exceed screening risk thresholds and thus might be considered for screening; and (5) compare the PLCO_m2012_ risks and lung cancer rates in high-risk PLCO smokers aged 54–64 y versus ≥65–80 y.

## Methods

### Design Overview, Setting, and Participants

PLCO and NLST study designs and results have been described previously [1],[17]–[21]. For both trials, institutional review board approvals were obtained at all study centers, and written informed consent was obtained from all participants. In this study we use PLCO (control arm *n = *77,455, CXR arm *n = *77,445) and NLST (CXR arm *n = *26,730, CT arm *n = *26,722) data. This study included histologically confirmed lung cancers and lung cancer deaths, which were identified from medical record reviews, death certificates, and National Death Index retrieval, and a death review committee classified causes of death. Predictor variable data were collected through epidemiological questionnaires administered at baseline.

### Statistical Analysis

We determined two different thresholds for using PLCO_m2012_ risk to identify screening candidates. For the first, we determined the PLCO_m2012_ risk threshold above which lung cancer mortality rates in the NLST CT arm appear to be consistently lower than those in the NLST CXR arm (named T_PLCOm2012_). This yields a threshold above which there is reliable evidence of mortality benefit.

As an alternative threshold, we applied the USPSTF criteria to the PLCO intervention (CXR) arm smokers and estimated the proportion of the cohort that would be selected for screening. We then found the PLCO_m2012_ risk threshold that identified a proportion of smokers equivalent to that of the USPSTF criteria (named T_USPSTF_). If one planned to screen the same proportion of the population as recommended by the USPSTF but using PLCO_m2012_ risk to select screenees, then it seems reasonable to use T_USPSTF_.

For T_PLCOm2012_ and T_USPSTF_, we estimated the sensitivity, specificity, and positive predictive values (PPVs) for lung cancer incident cases and deaths using PLCO intervention arm participants, and compared them to those observed for the USPSTF criteria. Because the PLCO_m2012_ model was developed in the PLCO control arm smokers, using the PLCO intervention arm smokers for comparisons with USPSTF criteria made for fairer comparisons. Although the PLCO age criteria for enrollment were similar to those in the NLST (ages 55 to 74 y inclusive), 14,678 of 40,447 (36.3%) of PLCO intervention arm smokers with exit time data contributed follow-up times for age range 75–80 y (USPSTF age criteria difference from NLST criteria), and these data were included in this analysis, making possible some evaluation of the USPSTF criteria.

The PLCO_m2012_ model was developed using lung cancer incidence occurring in 6 y of follow-up so as to make it applicable to NLST participants, the majority of whom had 6 y of follow-up but not much more. In the current study of lung cancer incidence, we truncated follow-up to 6 y. To adequately evaluate the impact of lung cancer on mortality, we extended the follow-up in the PLCO for an additional 5 y. All PLCO lung cancer deaths in 11 y of follow-up were studied. In the PLCO, 99.9% of lung cancer deaths were preceded by a documented lung cancer diagnosis.

To guide interpretation of risk values, we produced kernel density plots (“smoothed histograms” prepared using the Epanechnikov function [22]) that describe the distributions of PLCO_m2012_ risks in a variety of groups.

To determine whether high-risk never-smokers exceed our screening risk thresholds, we could not use the PLCO_m2012_ model because it was prepared for smokers. We prepared a model analogous to the PLCO_m2012_ model that included never-smokers. The resulting model, PLCO_all2014_, was validated using the PLCO intervention arm data by assessing the area under the receiver operator characteristic curve (AUC) and assessing calibration by plotting observed and predicted probabilities by deciles of model risk. Additionally, we assessed calibration by evaluating the median and 90th percentiles of absolute error between model-predicted probability and observed probability, where the latter was estimated from a lowess (locally weighted scatterplot smoothing) plot of lung cancer versus risk [23],[24]. Model calibration was further evaluated by Cox recalibration (synonym logistic recalibration) in PLCO intervention arm (validation) data. This method evaluates the amount of adjustment that is required in the intercept and beta coefficient of the original model logits (log odds) when predicting lung cancer using the original model logits in a logistic regression model in validation data [25],[26].

For the AUCs and summary statistics for absolute errors, 95% confidence intervals were estimated using bias-corrected percentile intervals in 1,000 bootstrap re-samplings [27]. Bootstrap samples were the same size as the original estimation sample, and sampling was done with replacement.

New studies might want to evaluate differences in the efficiency of screenee sample selection by applying both the USPSTF and PLCO_m2012_ criteria to enroll individuals. We produced sample size calculations for finding significant differences between the USPSTF and PLCO_m2012_ criteria in the proportion of individuals selected for screening, the proportion of lung cancers detected, and PPV. Sample size calculations were based on two-sample paired proportions and large-sample McNemar's test [28].

Confidence intervals and *p*-values were prepared using methods described by Brown and colleagues [29] and by Miettinen [30] for tests of proportions and rates, respectively. To test for a difference in a skewed continuous variable between two groups, we used a non-parametric test of trend [31]. To test for a difference in continuous variables with roughly normal distributions between two groups, we used Student's *t*-test not assuming equal variances, and to test for differences in proportions, we used the chi-square test. The number needed to screen (NNS) to prevent one lung cancer death and 95% confidence intervals were prepared by methods described by Bender [32]. For all hypothesis testing, we used two-sided *p*-values <0.05. Statistics were prepared using Stata 13.1 MP (StataCorp, College Station, Texas).

## Results

The study populations and PLCO_m2012_ model and its performance statistics have been described previously [11], and the PLCO_m2012_ model is summarized in Table S1.

### Risk Threshold for Screening Selection

**Figure 1:**
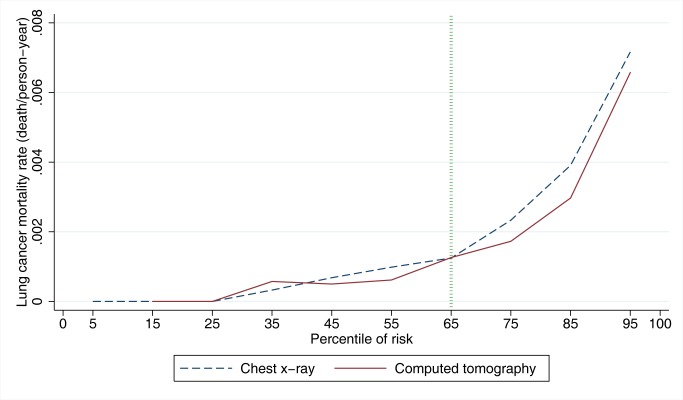
Lung cancer mortality rates in NLST arms by PLCO_m2012_ model risk deciles. PLCO_m2012_ model risk decile boundaries were established in PLCO control smokers. PLCO_m2012_ is the lung cancer risk prediction model described in [11].

Lung cancer mortality rates by NLST intervention arm and by decile of PLCO_m2012_ risk are presented in Figure 1 and Table 1. PLCO_m2012_ decile cutpoints were based on PLCO control arm smokers, not the NLST sample, which is unrepresentative of the general population because it was selected to comprise high-risk individuals. Consistently lower lung cancer mortality for CT-screened NLST participants compared to CXR-screened participants is observed in the eighth, ninth, and tenth PLCO_m2012_ risk deciles. According to the mortality rate ratio and rate difference for CT versus CXR, at the midpoint of the seventh decile there is no strong effect in either direction (Table 1). In the fourth, fifth, and sixth deciles, two estimates suggest CT screening has a protective effect, and one estimate suggests no protective effect. For these three deciles, findings are inconsistent, and because estimates are based on only 46 deaths in six trial arm–decile strata, no firm conclusions can be drawn. In the first three deciles, there are no lung cancer deaths in either trial arm. The lung cancer mortality reduction (rate difference) for CT versus CXR in the NLST in the 30th to <65th percentile risk range is small and not statistically significant: 1.60 per 10,000 person-years of follow-up (95% CI –1.96 to 5.16, *p = *0.38), and in the 65th to 100th percentile risk range is 4-fold larger and is statistically significant: 6.43 per 10,000 person-years of follow-up (95% CI 1.53 to 11.33, *p = *0.010). Although the CT minus CXR mortality difference was not statistically significant in any single decile of risk, such comparisons were not expected to be significant, as the statistical power of small subset analyses is limited, and the analyses were not designed for independent hypothesis testing.

**Table 1:** Mortality rates, rate ratios, and rate differences in NLST participants by trial arm and by decile of PLCO_m2012_ risk.

Category	Risk Percentile									
	0–10	>10 to 20	>20 to 30	>30 to 40	>40 to 50	>50 to 60	>60 to 70	>70 to 80	>80 to 90	>90 to 100
**CXR arm**										
Number of individuals in decile group	1	6	87	471	1,566	2,958	4,320	5,378	5,428	5,748
Number of deaths	0	0	0	1	7	19	35	81	135	254
Person-years of follow-up	6.67	38.21	555.29	3,062.57	10,274.54	19,309.86	28,065.96	34,721.42	34,543.86	35,454.52
Lung cancer mortality per 10,000 person-years	0	0	0	3.27	6.81	9.84	12.47	23.33	39.08	71.64
**CT arm**										
Number of individuals in decile group	0	2	67	526	1,528	2,982	4,261	5,441	5,569	5,626
Number of deaths	0	0	0	2	5	12	35	61	106	230
Person-years of follow-up	0	13.71	427.03	3,484.44	10,005.65	19,479.05	27,763.18	35,323.81	35,690.19	35,004.87
Lung cancer mortality per 10,000 person-years	NA	0	0	5.74	5.00	6.16	12.61	17.27	29.70	65.71
Rate ratio (CT mortality/CXR mortality)	NA	NA	NA	1.76	0.73	0.62	1.01	0.74	0.76	0.92
**Rate ratio 95% CI**	NA	NA	NA	0.09 to 103.71	0.18 to 2.68	0.28 to 1.36	0.61 to 1.66	0.52 to 1.04	0.58 to 0.99	0.76 to 1.10
**Rate difference per 10,000 person-years** * **(CT mortality − CXR mortality)**	NA	0	0	2.48	−1.82	−3.68	0.14	−6.06	−9.38	−5.94
**Rate difference 95% CI**	NA	0–0	0–0	−7.74 to 12.68	−8.50 to 4.87	−9.31 to 1.95	−5.74 to 6.01	−12.74 to 0.62	−18.07 to −0.70	−18.17 to 6.30

PLCO_m2012_ model risk decile boundaries were established in PLCO control smokers.

*Rate difference is incidence rate in CT arm per 10,000 minus incidence rate in CXR arm per 10,000. A negative absolute rate indicates a lower rate of lung cancer death in the CT arm compared to the CXR arm. PLCO_m2012_ refers to the lung cancer risk prediction model described in [11].

NA, not applicable (because of zero occurring in denominator).

The NNS to prevent one lung cancer death in the 65th to 100th percentile risk group is 255 (95% CI 143 to 1,184), which is statistically significant, and is a 25% improvement over the NNS of 320 reported for the NLST as a whole [1]. The NNS in the 30th to <65th percentile risk group is 963 (95% CI 291 to −754), which is not statistically significant. The NNS could not be calculated in the <30th percentile risk group because no lung cancer deaths were observed.

**Figure 2:**
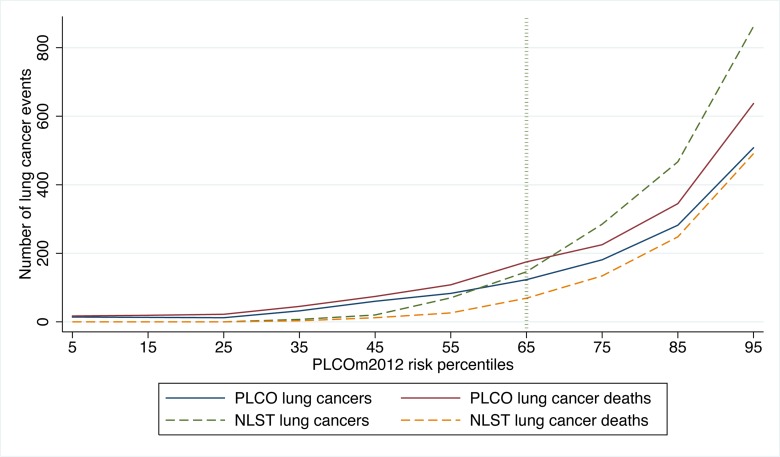
Number of lung cancer cases and deaths in PLCO and NLST by PLCO_m2012_ percentiles of risk. PLCO and NLST lung cancer cases and NLST lung cancer deaths were identified in 6 y of follow-up, and PLCO lung cancer deaths were identified in 11 y of follow-up. Calculations were based on PLCO_m2012_ deciles of risk, and the percentiles shown are the midpoints of each decile range. PLCO_m2012_ refers to the lung cancer risk prediction model described in [11].

**Figure 3:**
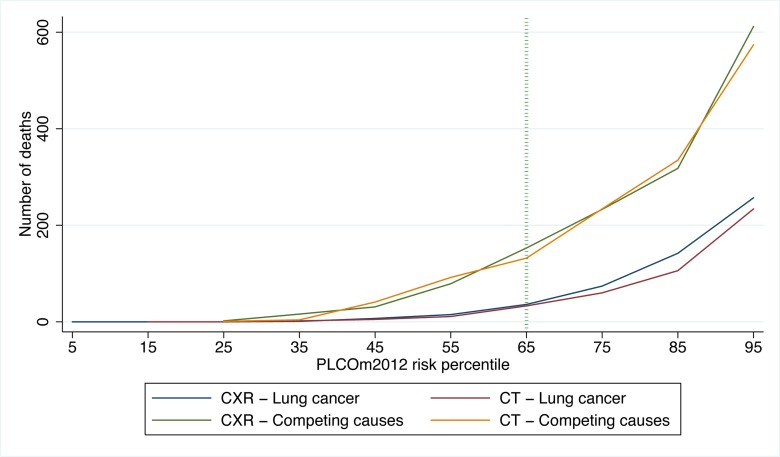
NLST deaths from lung cancer and competing causes by trial arm and decile of PLCO_m2012_ risk. CT is the LDCT screening arm; CXR is the CXR screening arm. PLCO_m2012_ refers to the lung cancer risk prediction model described in [11].

In PLCO smokers, the PLCO_m2012_ 65th percentile represents a risk of 0.0151 (95% CI 0.0149 to 0.0153) (T_PLCOm2012_), and for this threshold the sensitivity, specificity, and PPV for lung cancer incidence in 6 y are 80.9% (95% CI 78.6%–83.0%), 65.9% (95% CI 65.5%–66.2%), and 4.1% (95% CI 3.9%–4.3%), respectively, and for lung cancer mortality in 11 y are 78.6% (95% CI 76.5%–80.5%), 66.0% (95% CI 65.7%–66.4%), and 5.1% (95% CI 4.8%–5.3%), respectively. The PLCO_m2012_ risk ≥0.0151 threshold captures most but not all lung cancer cases and deaths in the PLCO and NLST (Figure 2). Figure 3 demonstrates that (1) the number of competing causes of death does not differ substantially between the NLST arms, (2) competing causes of death are substantially greater in number than lung cancer deaths, (3) elevated risks of competing causes of death start occurring around the 35th percentile of PLCO_m2012_ model risk, and (4) beneficial screening effects (mortality reductions) are present in the highest three deciles, notwithstanding high competing risks.

### USPSTF versus PLCO_m2012_ Risk ≥0.0151 Criteria for Selecting Screenees

When the USPSTF and PLCO_m2012_ risk ≥0.0151 criteria were applied to the PLCO intervention arm smokers (*n* = 37,327), 20,712 individuals (55.5%) were classified as negative (not selected for screening) by both approaches, and 10,475 (28.1%) individuals were classified as positive (selected for screening) by both criteria (Table 2, cells a and d). The discordant classifications are informative (Table 2, cells b and c). Compared to the USPSTF criteria, if the PLCO_m2012_ risk ≥0.0151 criterion were applied to select individuals for screening in PLCO intervention arm smokers, 8.8% (12,920 versus 14,170, *p<*0.001) fewer individuals would be selected, and 12.4% (542 versus 482, *p<*0.001) more lung cancers would be detected (Table 2). For identifying lung cancer cases, PLCO_m2012_ risk ≥0.0151 and USPSTF criteria sensitivities were 80.1% (95% CI 76.8%–83.0%) versus 71.2% (95% CI 67.6%–74.6%) (*p<*0.001), specificities were 66.2% (95% CI 65.7%–66.7%) versus 62.7% (95% CI 62.2%–63.1%) (*p<*0.001), and PPVs were 4.2% (95% CI 3.9%–4.6%) versus 3.4% (95% CI 3.1%–3.7%) (*p<*0.001), respectively.

**Table 2:** Distribution of observations and lung cancer events by USPSTF criteria and PLCO_m2012_ risk ≥0.0151 criterion status in PLCO intervention arm smokers.

PLCO_m2012_ risk	USPSTF Criteria Negative	USPSTF Criteria Positive	Total
PLCO_m2012_ risk ≥0.0151 negative	*n = *20,712 (cell percent = 55.5%)Lung cancers = 101Lung cancer deaths = 141(a)	***n = *** **3,695 (cell percent = 9.9%)** **Lung cancers = 33** **Lung cancer deaths = 48** **(b)**	*n = *24,407 (column percent = 65.4%)Lung cancers = 135Lung cancer deaths = 189
PLCO_m2012_ risk ≥0.0151 positive	***n = *** **2,445 (cell percent = 6.6%)** **Lung cancers = 93** **Lung cancer deaths = 102** **(c)**	*n = *10,475 (cell percent = 28.1%)Lung cancers = 449Lung cancer deaths = 554(d)	*n = *12,920 (column percent = 34.6%)Lung cancers = 542Lung cancer deaths = 656
Total	*n = *23,157 (row percent = 62.0%)Lung cancers = 195Lung cancer deaths = 243	*n = *14,170 (row percent = 38.0%)Lung cancers = 482Lung cancer deaths = 602	*N = *37,327 (cell percent = 100%)Lung cancers = 677Lung cancer deaths = 845

Bold indicates informative cells in which disagreement exists between the two classification criteria. PLCO_m2012_ refers to the lung cancer risk prediction model described in [11].

**Figure 4:**
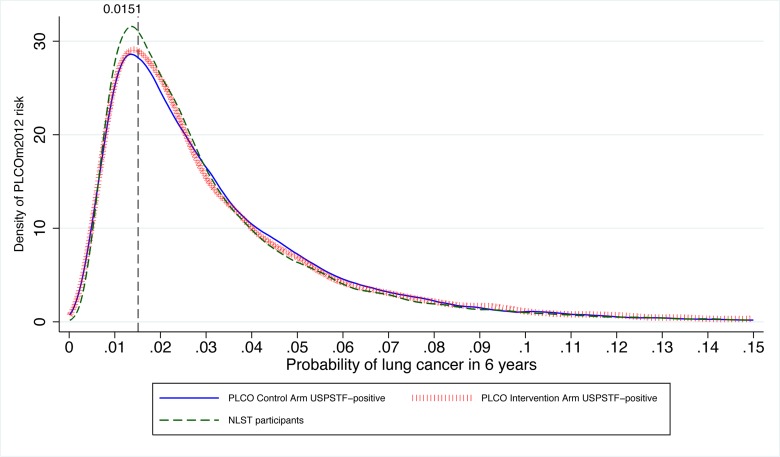
Distribution of PLCO_m2012_ risks in PLCO ever-smokers who are USPSTF-criteria-positive or are NLST participants. The vertical line indicates the PLCO_m2012_ risk ≥0.0151 threshold. The graph is right-truncated. PLCO_m2012_ is the lung cancer risk prediction model described in [11].

Many USPSTF-criteria-positive PLCO smokers and NLST participants had risks below the PLCO_m2012_ risk ≥0.0151 threshold (Figure 4). Of NLST participants and USPSTF-criteria-positive PLCO intervention arm participants, 26.6% and 26.1% had PLCO_m2012_ risks below 0.0151, respectively. For example, individuals who are age 55 y, have a graduate degree, have body mass index of 32 kg/m^2^, have no personal history of cancer, have no family history of lung cancer, do not have chronic obstructive pulmonary disease, are white, and are former smokers who quit smoking 14 y ago and smoked on average 20 cigarettes a day for 30 y have a 6-y lung cancer risk of 0.004, or 4 in 1,000, but would meet NLST/USPSTF criteria for CT screening. Other scenarios can be explored using the risk calculator available at http://www.brocku.ca/lung-cancer-risk-calculator.

**Figure 5:**
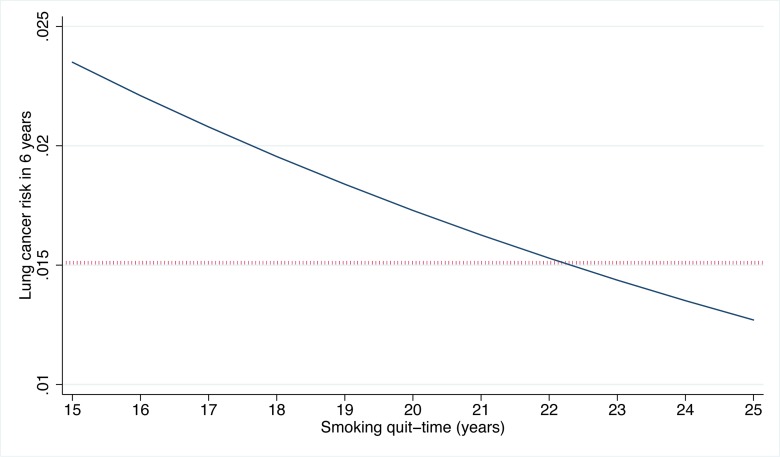
PLCO_m2012_-estimated risks for high-risk individuals by smoking quit time in former smokers. Estimates were prepared for white former smokers who are 68 y old, are high-school graduates, have a body mass index of 27 kg/m^2^, have no family history of lung cancer, have no personal history of cancer, started smoking at age 14 y, and smoked on average 30 cigarettes per day. As the quit time increases, smoking duration correspondingly decreases. The dotted horizontal line indicates the PLCO_m2012_ ≥0.0151 risk threshold. PLCO_m2012_ refers to the lung cancer risk prediction model described in [11].

The USPSTF recommends that lung cancer screening stop once an individual's smoking quit time exceeds 15 y. The PLCO_m2012_ model demonstrates that some high-risk individuals can remain at elevated risk that justifies screening well past 15 y after cessation (Figure 5). Of the 35,897 PLCO smokers who had smoking quit time >15 y, 3,064 (8.5%) met the PLCO_m2012_ risk ≥0.0151 threshold for screening, and of these 89, or 2.9%, had lung cancer diagnosed in 6 y of follow-up.

### USPSTF Criteria Risk Equivalent

Of 37,327 PLCO intervention arm smokers, 14,170 (38.0%) were USPSTF-criteria-positive. To select the same proportion of smokers based on highest PLCO_m2012_ risk—the alternate threshold we determined—requires a threshold at the 62.0th percentile of risk, a PLCO_m2012_ risk of 0.0134 (T_USPSTF_). Comparing screenee selection by this PLCO_m2012_ risk ≥0.0134 threshold and USPSTF criteria in PLCO intervention arm smokers for detecting lung cancer, the sensitivities were 83.2% (95% CI 80.1%–85.9%) versus 71.2% (95% CI 67.6%–74.6%) (*p<*0.001), specificities were 62.9% (95% CI 62.4%–63.4%) versus 62.7% (95% CI 62.2%–63.1%) (*p = *0.38), and PPVs were 4.0% (95% CI 3.7%–4.3%) versus 3.4% (95% CI 3.1%–3.7%) (*p<*0.001), respectively. Screenee selection based on the PLCO_m2012_ T_USPSTF_ is superior to screenee selection based on USPSTF criteria in all performance categories measured.

### Calibration at Risk Thresholds

If the PLCO_m2012_ risk ≥0.0151 or PLCO_m2012_ risk ≥0.0134 thresholds are to be used for selecting screenees, it is important that model calibration is high around these risks. At risks from 0.0100 to 0.0185 inclusive, the median, mean, and 90th percentiles of absolute error between observed and predicted risks were 0.00194 (95% CI 0.00194–0.00195), 0.00156 (95% CI 0.00154–0.00157), and 0.00210 (95% CI 0.00209–0.00210), respectively, suggesting reasonable calibration for decision-making risks. For example, for a model-predicted risk of 0.0151, we expect on average the calibration-corrected model-predicted risk to be 0.0151±0.00156, or between 0.0135 and 0.0167.

### Distributions of Risks

**Figure 6:**
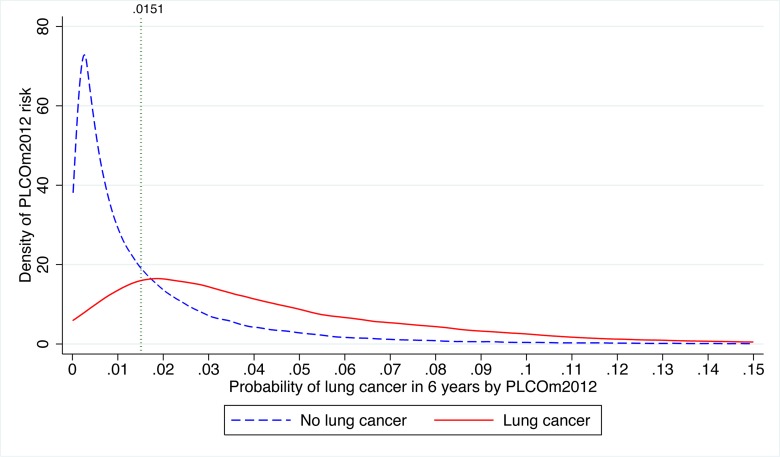
Distribution of PLCO_m2012_ risk in PLCO intervention arm smokers with and without lung cancer diagnosed in 6 y of follow-up. The risk threshold *p = *0.0151 is indicated by the vertical line. The graph is right-truncated. PLCO_m2012_ is the lung cancer risk prediction model described in [11].

The distributions of PLCO_m2012_ risks in PLCO intervention arm smokers by lung cancer status are presented in Figure 6. The PLCO_m2012_ risk ≥0.0151 threshold lies close to the intersection point above which the density of risk is greater in individuals diagnosed with lung cancer than in those not diagnosed with lung cancer. Of the 1,307 PLCO smokers who had lung cancer diagnosed during 6 y of follow-up, 250 (19.1%) had risks <0.0151, 1,025 (78.4%) had risks in the range 0.0151–0.1500 inclusive, and 32 (2.5%) had risks >0.1500; further, 221 (16.9%) had risks <0.0134 (T_USPSTF_), and 1,054 (80.6%) had risks in the range 0.0134–0.1500 inclusive.

Of the 1,667 PLCO smokers who died due to lung cancer during 11 y of follow-up, 357 (21.4%) had risks <0.0151, 1,279 (76.7%) had risks between 0.0151 and 0.1500 inclusive, and 31 (1.9%) had risks >0.1500; 300 (18.0%) had risks <0.0134 (T_USPSTF_), and 1,336 (80.1%) had risks between 0.0134 and 0.1500. In conclusion, whether using T_USPSTF_ or T_PLCOm2012_, the large majority of lung cancer cases and deaths occur between the risk threshold and a risk of 0.1500.

### Never-Smokers' Risk Model

A model analogous to the PLCO_m2012_ was developed in PLCO control arm never- and ever-smokers, and was validated in the PLCO intervention arm. The model, *PLCO_all2014_*, is described in Table S1. PLCO_all2014_ demonstrated high discrimination in the PLCO intervention group (validation data) (AUC  = 0.848, 95% 0.833–0.861). The median and 90th percentiles of absolute errors in the PLCO control and intervention arms, and PLCO intervention arm smokers and never-smokers, were all 0.0014 or smaller. Cox recalibration analysis found that the PLCO_all2014_ and the PLCO_m2012_ intercepts and original model logits (log odds) were overestimated slightly when evaluated in the PLCO intervention arm data, but none of the differences were statistically significant (Table S1). However, the PLCO_all2014_ logit was overestimated by 5.6%, and the test of whether this value differed from zero approached significance (*p = *0.0502). Figure S1 presents the mean observed and predicted risks by decile of PLCO_all2014_ predicted risk in PLCO participants. Figures S2 and S3 present the relationship between observed and predicted probabilities and absolute errors as they change with risk in PLCO control and intervention arm participants. These figures demonstrate that PLCO_all2014_ calibration is good for risks below 0.10, which include important decision-making thresholds. At risks above 0.15, the model overestimates risks. However, only a very small proportion of the population falls into this high-risk group (0.2% of PLCO participants had risks >0.15), and these individuals would be selected for screening based on elevated risk regardless.

Simple-to-use spreadsheet calculators for PLCO_m2012_ and PLCO_all2014_ are available at http://www.brocku.ca/lung-cancer-risk-calculator.

### Maximum Risks in Never-Smokers

According to the PLCO_all2014_ model, the theoretical maximum possible 6-y lung cancer risk in never-smokers is 3.5%. This model ceiling risk is estimated for a never-smoker who is 80 y, has not graduated from high school, has a body mass index of 18 kg/m^2^, is African-American, has chronic obstructive pulmonary disease, has a personal history of cancer, and has a family history of lung cancer. This theoretical maximum exceeds our screening thresholds. However, this combination of risk factors is expected to be rare. The maximum PLCO_all2014_ risk observed in 65,711 PLCO never-smokers was 0.0147, which is below our recommended PLCO_m2012_ risk ≥0.0151 threshold for lung cancer screening.

### Lung Cancer Risk and Incidence in Smokers Stratified by Age Dichotomized at 65 y

**Figure 7:**
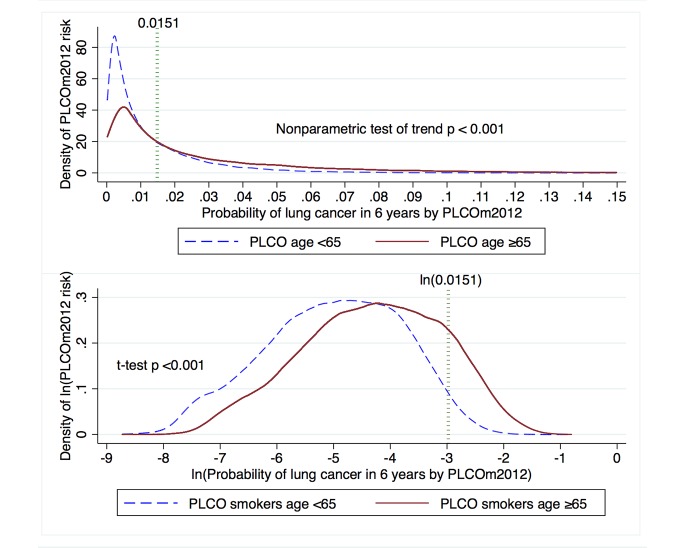
Distribution of PLCO_m2012_ risk and natural log-transformed risk in PLCO participants stratified by age dichotomized at 65 y. The PLCO_m2012_ risk ≥0.0151 threshold is marked by the dotted vertical line. The upper graph is right-truncated. PLCO_m2012_ is the lung cancer risk prediction model described in [11].

Because reimbursement for LDCT lung cancer screening may be provided to those 55–64 y of age through the Patient Protection and Affordable Care Act, and may not be provided by Medicare for those ≥65–80 y of age, we stratified analysis of risks and lung cancer incidence by these age strata. PLCO_m2012_ risk, lung cancer cumulative incidence overall, lung cancer cumulative incidence in those who had PLCO_m2012_ risk ≥0.0151 , and PPV were all statistically significantly greater in the older age stratum (Table 3; Figure 7).

**Table 3:** Comparison of PLCO_m2012_ risk and incident lung cancer in age strata of PLCO smokers dichotomized at age 65 y.

Category	Age		*p*-Value*
	<65 y	≥65 y	
PLCO_m2012_ risk mean†	0.0067 (95% CI 0.0066–0.0068)	0.013 (95% CI 0.013–2.014)	*p<*0.001
Number of participants with PLCO_m2012_ risk ≥0.0151	13,691/48,560 (28.2%)	12,131/25,288 (48.0%)	*p<*0.001
Incident lung cancers in 6 y of follow-up	629/48,560 (1.3%)	679/25,288 (2.7%)	*p<*0.001
Incident lung cancers in 6 y of follow-up in participants with PLCO_m2012_ risk ≥0.0151 (PPV)	474/13,691 (3.5%)	583/12,131 (4.8%)	*p<*0.001

PLCO_m2012_ refers to the model described in [11], and described in Table S1.

*p-Value for PLCO_m2012_ risk was by *t*-test with unequal variance applied to natural-log-transformed risk values. *p*-Values for comparing proportions were by chi-square test.

† Because PLCO_m2012_ risk distributions are right-skewed, geometric means are presented.

## Discussion

Tammemägi et al. [11] and Kovalchik et al. [12] presented evidence supporting the idea that risk models are more efficient for selecting individuals for LDCT lung cancer screening than the NLST criteria. However, neither study indicated where a suitable risk threshold for selecting screenees might be. The current study demonstrates that the PLCO_m2012_ model with a PLCO_m2012_ risk ≥0.0151 threshold for selecting individuals for screening is statistically and clinically more efficient than the USPSTF criteria, because it leads to a smaller number of individuals being screened, identifies significantly more lung cancers, and has higher PPV. In PLCO intervention arm smokers, compared to the USPSTF criteria, the PLCO_m2012_ risk ≥0.0151 criterion selects an 8.8% smaller sample and detects 12.4% more lung cancers, and because specificity is significantly improved, fewer false-positive screens are expected. Based on PLCO smokers, to sample the same proportion for screening as is selected by USPSTF criteria, a PLCO_m2012_ risk ≥0.0134 threshold is required. Bach and Gould [33] and others have emphasized the importance of limiting screening to high-risk individuals [12],[33]. We recommend use of T_PLCOm2012_ over T_USPSTF_. Also, because the proportion of smokers at high risk will change over time [34], we discourage screening selection based on a flat proportion of smokers ranked by risk.

If selection of individuals for lung cancer screening is to use model-based risk thresholds, it is critical that the model is well calibrated near the threshold. Unlike the PLCO_m2012_ model, some prediction models demonstrate poor calibration. For example, the Liverpool Lung Project model's [35] overall expected/observed ratio indicates 24% calibration error, and around the 0.0151 threshold its calibration error is 57% (Appendix Figure 1 in [36]).

With regard to interpreting risk probabilities, it is important to appreciate that PLCO_m2012_ risks between 0.0151 and 0.1500 may not appear to be high in absolute terms, but are clinically important because the vast majority of lung cancers occur in this range.

Based on PLCO data and the PLCO_all2014_ model, analyses indicate that the general population of never-smokers should not be screened given our current state of knowledge. Although the PLCO_all2014_ model demonstrated that, in theory, the highest possible risks in never-smokers exceed our risk threshold, none of the large number of never-smokers in the PLCO had risks exceeding PLCO_m2012_ ≥0.0151.

Our analysis found that lung cancer risk, lung cancer cumulative incidence overall, and lung cancer cumulative incidence in those with PLCO_m2012_ risk ≥0.0151 were statistically significantly greater in PLCO smokers aged ≥65–80 y than in those aged 55–64 y. These findings are as expected, because the older age stratum had longer opportunity for exposure, it had additional risk contributed by age alone, and members belonged to cohorts with higher smoking rates. Findings reported by Kovalchik et al. [12] and in the current study (Table 1) indicate that the LDCT screening benefits for mortality reduction are greatest in high-risk individuals. This evidence argues that lung cancer screening should not be withheld from the older group of smokers.

Evaluation based on cost-effectiveness was not possible in this study because of absence of data. However, because the PLCO_m2012_ risk ≥0.0151 criterion for selecting screenees for lung cancer screening selects fewer screenees, and improves sensitivity and specificity, it should translate into improved cost-effectiveness over the USPSTF criteria. Risk factors, in particular smoking behavior, might have changed during study follow-up, but such changes were not included in the modeling, because high-quality and complete data on many important factors from the follow-up period were not available. Our study has strengths. This study was carried out using the PLCO_m2012_ and PLCO_all2014_ models, both of which were developed in a large prospectively followed population-based sample, and both models demonstrated high discrimination and calibration in validation data.

Use of the PLCO_m2012_ risk ≥0.0151 threshold for screening should result in more efficient and cost-effective screening programs. This should make lung cancer screening more attractive for policy-makers and more affordable for health systems. Some jurisdictions may not be able to afford lung cancer screening for the number of screenees that a PLCO_m2012_ risk ≥0.0151 threshold would indicate and may use higher thresholds. Use of extremely high-risk thresholds may have limitations. Figure 1 demonstrates that the screening effect does not continue to widen substantially beyond the 75th percentile. Figure 3 demonstrates that deaths from competing causes rise sharply with PLCO_m2012_ risk, increasing the probability of death in lung cancer screenees selected from the highest risk group. Furthermore, current heavy smokers are more likely to be non-participants, non-adherents, or dropouts [37]–[40].

Practical implementation of the PLCO_m2012_ model or similar models for selecting individuals for lung cancer screening need not be onerous. For example, the Pan-Canadian Early Detection of Lung Cancer Study was successful in identifying and recruiting individuals at high risk for lung cancer by applying a prototype of the PLCO risk prediction model, using a central, free 1-800 call-in number and a spreadsheet risk calculator to identify individuals who met study risk-level entry criteria [41]. Smart-phone apps, which will become available in the near future, will further improve the utility of complex, but accurate and valuable, prediction algorithms.

Given USPSTF recommendations, how can the PLCO_m2012_ risk ≥0.0151 criterion be implemented into lung cancer screening programs? One investigative approach is to enroll individuals into screening programs if they qualify by either USPSTF or PLCO_m2012_ risk criteria. This approach is justifiable because it should be more cost-effective than using the USPSTF criteria alone. With this program design, a sample size of 7,000 will have ≥0.80 power (alpha error  = 0.05) to identify clinically important differences in proportions selected for screening, proportions of lung cancers detected, and PPVs for PLCO_m2012_ risk ≥0.0151 versus USPSTF criteria. Findings from different centers can be pooled to quickly allow meta-analyses. Findings from such investigations can guide future selection procedures.

## Conclusions

Selection of individuals for LDCT lung cancer screening programs using the PLCO_m2012_ risk ≥0.0151 criterion should improve screening efficiency compared to selection by USPSTF criteria. Currently, never-smokers should not be screened. Lung cancer screening of high-risk older smokers (≥65–80 y) should be encouraged.

## Supporting Information

Figure S1 **PLCO_all2014_ calibration—observed and predicted 6-y lung cancer risk in the PLCO cohort based on decile of risk.** PLCO_all2014_ refers to the lung cancer risk prediction model described in Table S1. The PLCO_all2014_ model was developed using data on never- and ever-smokers in the PLCO control arm and is analogous to the PLCO_m2012_ model [11] with respect to predictors.
(EPS)

Figure S2 **PLCO_all2014_-model-predicted 6-y probabilities of lung cancer versus observed probabilities (line graphs) in PLCO control and intervention arm participants.** Distribution of lung cancer cases and non-cases in 6 y of follow-up by PLCO_all2014_ risk is presented in the scatter diagrams. To improve fit of graphs, they were truncated at risks of 0.2. Only 0.05% of PLCO participants had PLCO_all2014_ risk ≥0.2 (36 control arm and 37 intervention arm participants).
(EPS)

Figure S3 **PLCO_all2014_ model absolute error between predicted 6-y probabilities of lung cancer and observed probabilities (line graphs) in PLCO control and intervention arm participants.** Distribution of lung cancer cases and non-cases in 6 y of follow-up by PLCO_all2014_ risk is presented in the scatter diagrams. To improve fit of graphs, they were truncated at risks of 0.2. Only 0.05% of PLCO participants had PLCO_all2014_ risk ≥0.2 (36 control arm and 37 intervention arm participants).
(EPS)

Table S1 **Risk model predictors and predictive performance statistics for the PLCO_m2012_ and PLCO_all2014_ models.**
(PDF)

## Abbreviations

AUC: area under the receiver operator characteristic curve
CT: computed tomography
CXR: chest X-ray
LDCT: low-dose computed tomography
MEDCAC: Medicare Evidence Development & Coverage Advisory Committee
NLST: National Lung Screening Trial
NNS: number needed to screen
PLCO: Prostate, Lung, Colorectal and Ovarian Cancer Screening Trial
PPV: positive predictive value
USPSTF: U.S. Preventive Services Task Force
