# Correction: Evaluation of the Lung Cancer Risks at Which to Screen Ever- and Never-Smokers: Screening Rules Applied to the PLCO and NLST Cohorts

**DOI:** 10.1371/journal.pmed.1001787

**Published:** 2015-01-28

**Authors:** The PLOS Medicine Staff

S1 Table includes some incorrect odds ratios in the table and corresponding coefficients in the formula due to the inclusion of numbers from a penultimate version of the model. The corrected table can be viewed below.

## Supporting Information

S1 Table Risk model predictors and predictive performance statistics for the PLCOm2012 and PLCOall2014 models. (PDF)
