# Supplementary material for: Correction: Evaluation of the Lung Cancer Risks at Which to Screen Ever- and Never-Smokers: Screening Rules Applied to the PLCO and NLST Cohorts
Source: PLoS Med. 2015 Jan 28;12(1):e1001787. doi: 10.1371/journal.pmed.1001787 (PMC4309585; doi:10.1371/journal.pmed.1001787)
Supplement: S1 Table — (PDF) [file pmed.1001787.s001.pdf]

**Table S1**. Risk model predictors and predictive performance statistics for $PLCO_{m2012}$* and $PLCO_{all2014}$* models

| **Model** / **PLCO participants sampled** | **$PLCO_{m2012}$ ‡** Ever-smokers only | **$PLCO_{all2014}$ §** Never- & ever-smokers |
|---|---|---|
| **Variables** | **Odds Ratio (95% CI, p-value)** | **Odds Ratio (95% CI, p-value)** |
| Age † (per one year) | 1.081 (1.057 to 1.105; p < 0.001) | 1.083 (1.061 to 1.105; p < 0.001) |
| Race/ethnicity (self-reported) | | |
| White | Referent group | Referent group |
| Black | 1.484 (1.083 to 2.033; p = 0.014) | 1.379 (1.009 to 1.885; p = 0.044) |
| Hispanic | 0.475 (0.195 to 1.160; p = 0.102) | 0.440 (0.181 to 1.073; p = 0.071) |
| Asian | 0.627 (0.332 to 1.185; p = 0.150) | 0.592 (0.323 to 1.085; p = 0.090) |
| Native Hawaiian or Pacific Islander | 1 | 0.256 (0.036 to 1.838; p = 0.175) |
| American Indian or Alaskan Native | 2.793 (0.992 to 7.862; p = 0.052) | 2.593 (0.927 to 7.252; p = 0.069) |
| Education † (per 6 levels) | 0.922 (0.874 to 0.972; p = 0.003) | 0.916 (0.870 to 0.964; p = 0.001) |
| Body mass index † (weight in kilograms/height in meters squared) | 0.973 (0.955 to 0.991; p = 0.003 | 0.971 (0.954 to 0.989; p = 0.001) |
| Chronic obstructive pulmonary disease (yes vs. no) | 1.427 (1.162 to 1.751; p = 0.001) | 1.413 (1.154 to 1.730; p = 0.001) |
| Personal history of cancer (yes vs. no) | 1.582 (1.172 to 2.128; p = 0.003) | 1.623 (1.219 to 2.162; p = 0.001) |
| Family history of lung cancer (yes vs. no) | 1.799 (1.471 to 2.200; p < 0.001) | 1.796 (1.478 to 2.182; p < 0.001) |
| Smoking status (former=0, current=1) | 1.297 (1.047 to 1.605; p = 0.017) | Referent group |
| Former smoker (yes vs. no) | Not applicable | 12.711 (5.109 to 31.627; p < 0.001) |
| Current smoker (yes vs. no) | Not applicable | 16.440 (6.643 to 40.683; p < 0.001) |
| Smoking intensity (average cigarettes/day) | p < 0.001 ‡ | p < 0.001 § |
| Smoking duration (per year) | 1.032 (1.014 to 1.051; p = 0.001) | 1.031 (1.013 to 1.049; p = 0.001) |
| Smoking quit-time | 0.970 (0.950 to 0.990; p = 0.003) | 0.968 (0.949 to 0.988; p = 0.002) |
| Model constant | -4.532506 (unexponentiated) | -7.02198 (unexponentiated) |
| **Predictive Performance – Discrimination** | ROC AUC (95% CI) | ROC AUC (95% CI) |
| AUC in PLCO control (development) arm (ever- ± never-smokers) | 0.803 (0.782 to 0.813) (smokers only, n=36286) | 0.859 (0.845 to 0.872) (all, n=68706) |
| AUC in PLCO intervention (validation) arm (ever- ± never- smokers) | 0.797 (0.782 to 0.813) (smokers only, n=37,332) | 0.848 (0.833 to 0.861) (all, n=70540) |
| AUC in PLCO never-smokers in control & intervention arms | Not applicable | 0.662 (0.607 to 0.709) (n=65711) |
| **Predictive Performance – Calibration – Cox Recalibration in PLCO Intervention Arm** ‖ | | |
| Recalibrated factor added to adjust the original model intercept | -0.131 (-0.394 to 0.132; p = 0.14) ‖ | -0.138 (-0.361 to 0.084; p = 0.223) ‖ |
| Recalibration factor to multiply the original model logit | 0.946 (0.874 to 1.018; p = 0.33) ‖ | 0.944 (0.888 to 1.000; p = 0.0502) ‖ |
| **Predictive Performance – Calibration – Median & 90th percentile absolute error** | | |
| PLCO control (development) arm (ever- ± never-smokers) | 0.0087, 0.04 | 0.0002, 0.0005 |
| PLCO intervention (validation) arm (ever- ± never- smokers) | 0.0092, 0.04 | 0.0003, 0.0014 |
| PLCO never-smokers in control & intervention arms | Not applicable | 0.0002, 0.0003 |

***Abbreviations***: CI, confidence interval; n, number in subsample; PLCO, Prostate, Lung, Colorectal and Ovarian Cancer Screening Trial; ROC AUC, Receiver operator characteristic area under the curve.

* The $PLCO_{m2012}$ model was developed using PLCO control group ever-smokers' data and is described in Table S1 reference [1]. The $PLCO_{all2014}$ model was developed using PLCO control group never and ever-smokers' data and is analogous to the $PLCO_{m2012}$. Spreadsheet calculators for these two models are available from http://www.brocku.ca/lung-cancer-risk-calculator.

† Age is centered on 62 years, education is centered on level 4 out a possible 6, body mass index is centered on 27, and smoking duration was centered on 27 years, and smoking quit-time was centered on 10 years in PLCOm2012 and on 8.583417626 years in PLCOall2014. Education in six levels (1=less than high school completed; 2=high school graduate; 3=post high school training; 4=some college; 5=college graduate; 6=postgraduate or professional degree) is centered on the 4th level, some college.

Smoking intensity (average number of cigarettes smoked per day) had a nonlinear association described in the following equations.

‡To calculate PLCOm2012 6-year probability of lung cancer one must first calculate the LogitPLCOm2012 using the following formula:

$$\begin{aligned}
Logit = & -4.532506 + 0.0778868*(\text{Age} - 62) - 0.0812744*(\text{Education} - 4) - 0.0274194*(\text{BMI} - 27) + 0.3553063*(\text{COPD}) \\
& + 0.3944778*(\text{Black}) - 0.7434744*(\text{Hispanic}) - 0.466585*(\text{Asian}) + 0*(\text{Pacific Islander}) + 1.027152*(\text{Native American}) \\
& + 0.4589971*(\text{Personal history of cancer}) + 0.587185*(\text{Family history of lung cancer}) + .02597431*(\text{Smoking Status}) \\
& - 1.822606*\left[\left(\frac{\text{Average number cigarettes smoked per day}}{10}\right)^{-1} - 0.4021541613\right] \\
& + 0.0317321*(\text{Years smoked} - 27) - 0.0308572*(\text{Quit-time} - 10)
\end{aligned}$$

Then,

$$\text{6-year probability of lung cancer by PLCO}_{m2012} = \frac{e^{Logit_{PLCOm2012}}}{1+e^{Logit_{PLCOm2012}}}$$

§ To calculate $PLCO_{all2014}$ 6-year probability of lung cancer one must first calculate the $Logit_{PLCOall2014}$ using the following formula:

$$Logit_{PLCOall2014} = -7.02198 + 0.079597*(\text{Age} - 62) - 0.0879289*(\text{Education} - 4) - 0.028948*(\text{BMI} - 27) + 0.3457265*(\text{COPD})$$
$$+ 0.3211605*(\text{Black}) - 0.8203332*(\text{Hispanic}) - 0.5241286*(\text{Asian}) - 1.364379*(\text{Pacific Islander}) + 0.952699*(\text{Native})$$
$$+ 0.4845208*(\text{Personal history of cancer}) + 0.5856777*(\text{Family history of lung cancer})$$
$$+ 2.542472*(\text{Former Smoker})^{\blacklozenge} + 2.799727*(\text{Current Smoker})^{\blacklozenge} + (-0.1815486*\left[\left(\frac{\text{Average number cigarettes smoked per day}}{100}\right)^{-1} - 4.021541613\right])^{\blacklozenge}$$
$$+ 0.0305566*(\text{Years smoked} - 27)^{\blacklozenge} - 0.0321362*(\text{Smoking Quittime} - 8.593417626)^{\circ}$$

♦ Term applies only in ever-smokers. In never-smokers, reverts to zero.

° Term applies only to former-smokers. Othewise reverts to zero.

Then,

$$\text{6-year probability of lung cancer by } PLCO_{all2014} = \frac{e^{Logit_{PLCOall2014}}}{1 + e^{Logit_{PLCOall2014}}}$$

‖ Cox recalibration or logistic recalibration[2,3] was carried out by to determine what adjustments to the original model were required to fit the data in the validation data (PLCO intervention arm). If calibration was perfect and no adjustment is required, zero would be added to the intercept and the beta coefficient for the original model logit would be 1. The presented p-value tests if the intercept = 0 and beta coefficient = 1.

## Table S1 References


1. Tammemagi MC, Katki HA, Hocking WG, Church TR, Caporaso N, et al. (2013) Selection criteria for lung-cancer screening. The New England journal of medicine 368: 728-736.
2. Cox DR (1958) Two further applications of a model for binary regression. Biometrika 45: 562-565.
3. Woodward M (2014) Epidemiology : study design and data analysis. 3rd Edition. Boca Raton: CRC Press. xxii, 832 pages p.
